# Matured compost amendment improves compost nutrient content by changing the bacterial community during the composting of Chinese herb residues

**DOI:** 10.3389/fmicb.2023.1146546

**Published:** 2023-03-16

**Authors:** Xiuchao Song, Chao Lu, Jia Luo, Xin Gong, Dejie Guo, Yan Ma

**Affiliations:** ^1^Key Laboratory of Saline-Alkali Soil Improvement and Utilization (Coastal Saline-Alkali Lands), Ministry of Agriculture and Rural Affairs, Institute of Agricultural Resources and Environment, Jiangsu Academy of Agricultural Sciences, Nanjing, China; ^2^National Agricultural Experimental Station for Agricultural Environment, Luhe, Nanjing, China; ^3^Jiangsu Key Laboratory for Solid Organic Waste Utilization, Jiangsu Collaborative Innovation Center for Solid Organic Waste Resource Utilization, College of Resources and Environmental Sciences, Nanjing Agricultural University, Nanjing, China

**Keywords:** Chinese herb residue, matured compost, nitrogen and humic acid, bacterial community, co-occurrence network

## Abstract

Composting is a sustainable strategy to deal with organic waste. Our research aimed to study the influence of an amendment of 10% matured compost (MC) during Chinese herb residue (CHR) compost. Here, a 60-day CHR compost was performed, and MC application was able to reduce the nitrogen loss and enhance the humic acid accumulation during the composting as compared with the non-inoculated control (NC), by 25 and 19%, respectively. Furthermore, the matured compost amendment improved the diversity of the bacterial community, increased the complexity of the co-occurrence network, and changed the keystone and module hub bacteria during composting. The increased abundance levels of *Thermopolyspora*, *Thermobispora*, and *Thermosporomyces*, which were significantly higher in MC than in NC, may contribute to the degradation of cellulose and the formation of humic acid. Overall, this study extends our understanding of the effects of matured compost reflux on compost quality and the bacterial community.

## 1. Introduction

Traditional Chinese medicine is an essential part of the medical system in China. It has been extensively used in other countries and considered as a potential treatment method. With the global prevalence of traditional Chinese medicine and the development of the Chinese herb industry, massive amounts of Chinese herb residues (CHRs) have been produced (Zhao and Zhou, 2016; Kong et al., 2019). In China, which is the largest consumer and producer of herbs, more than 1.5 million tons of CHRs are produced every year without effective treatment (Wang et al., 2018). Due to the characteristics of high humidity and content of lignin and cellulose, there are many problems in CHR degradation and utilization (Huang et al., 2021). At present, a great number of CHRs are directly disposed of by burning, burying, and stacking, which can cause environmental pollution (Zeng et al., 2016; Zhou et al., 2016). Furthermore, organic-rich CHRs without effective treatment are a waste of resources and restrict the sustainable development of traditional Chinese medicine, accompanied by possible social problems (Zhou et al., 2018).

The major ways of utilizing CHRs are for ingredient re-extraction, organic acid production, composting, etc. (Kong et al., 2019). Chinese herb residues are often used as an additive in the composting process to participate in the composting of other agricultural and forestry wastes, such as animal manure, food waste, etc. (Kong et al., 2019). Previous research indicated that CHRs could also be used to extract bioactive substances or produce organic acids: Wang et al. (2018) extracted succinic acid from six different herbs, while Zheng et al. (2017) found that co-fermentation of *Sophora flavescens* residues and food waste could increase the production of L-lactic acid. However, compared with the traditional organic acid production method, the economic benefit and yield of organic acid produced by CHR composting are insufficient (Tao et al., 2021). Meanwhile, due to the complexity of traditional Chinese medicine, it is difficult to design a unified method for the extraction of active substances and the production of organic acids (Tao et al., 2021). Pyrolysis and gasification have also been reported for the treatment of CHRs; however, these two methods ignore the nutrient elements in CHRs and may consume a large amount of energy (Wang et al., 2010; Zeng et al., 2016; Shang et al., 2017). Thus, composting was recognized as an economical and environmentally friendly method to deal with CHRs (Li et al., 2017). However, few studies have focused on the composting of CHRs, and even fewer studies have examined whether recycling of matured compost can accelerate the maturation of CHR compost.

During the composting process, microorganisms play a vital role in decomposition of macromolecules, and the appearance of some microorganisms reflects the quality of composting (Gajalakshmi and Abbasi, 2008; Bhatia et al., 2013). The bioactive constituents remaining in the CHRs may affect microbial activity in the composting pile and may thus influence the final compost quality (Greff et al., 2021). Meanwhile, the high lignin and cellulose contents of CHRs make it difficult for them to be decomposed and utilized by microorganisms (Akhtar et al., 2014). Lignocellulose in plants is the uppermost limiting factor influencing the maturation process during agricultural waste composting (Greff et al., 2021). The inoculation of different exogenous microbes is regarded as an effective method to degrade lignocellulose, but competition among the exogenous and native microbes may lead to uncertain results. Matured compost contains many mesophilic and thermophilic microbes which have a strong tolerance to the harsh environment and can live in harmony with native microbes during the composting process (Kato and Miura, 2008; Liu et al., 2020; Wang et al., 2021). Amendment with matured compost has been proven to reduce greenhouse gas emissions during the composting process of livestock manure and food waste, and to accelerate the maturation and humification of compost (Ma et al., 2019). However, the underlying biological mechanism is poorly studied. Due to their quick reproduction, high organic matter utilization efficiency, and high-temperature tolerance, bacterial species in composting have been studied extensively (de Gannes et al., 2013). Hence, it is worth studying whether the native bacterial community existing in the matured compost can be used to accelerate the composting process and improve the compost quality, in addition to examining its influence on the microbial community.

Therefore, we designed a 60-day experiment, and the purpose of this study was to investigate the influence of matured compost on CHR composting. The composting temperature, fulvic acid, humic acid, etc., were detected. The bacterial community was detected and analyzed by high-throughput sequencing. The overall objectives of our research were to: (1) explore the effects of matured CHR compost on the physical and chemical properties of compost; (2) investigate the bacterial community changes during composting; and (3) clarify the underlying biological mechanisms of matured compost promoting the composting process.

## 2. Materials and methods

### 2.1. Experiment design and composting conditions

Fresh Chinese herb residues was taken from Kangyuan Pharmaceutical Co., Ltd., Lianyungang, Jiangsu province. Chinese herb residues formula included artemisia annua, honeysuckle, poria cocos, peony bark, cassia twig, root of Rhizoma Polygoni, scallop, etc. Due to the high moisture content of the raw materials, the moisture content was controlled below 65% through the drying method. In order to ensure the temperature rise during the drying process, the height of the dried materials should not exceed 20 cm. Matured compost was a product that uses the same raw materials in the early stage, and the composting time is 4 months. The composting experiment was conducted at Haolaisi Fertilizer Co., Ltd., Lianyungang, Jiangsu province. There were three treatments in this experiment: non-inoculated control (NC), amendment of 10% matured compost (MC), and amendment of 10% sterilized matured compost (SC).

The initial dry matter weight of each treatment compost was about 3,000 kg, and the stacking length, width, and height were 18, 1.5, and 1.5 m, respectively. It was dumped according to the change in stack temperature, and the whole stacking time was maintained for about 60 days. Samples were collected on Days 1, 7, 21, 41, and 61 of composting as follows: The whole stack was divided into three sections, 6 points were selected for each section, and 200 g samples were taken from the outer, middle, and inner layers at each point, then mixed evenly. These samples were transported back to the laboratory in a low-temperature freezer with dry ice for DNA exaction, and then they were air-dried and ground for the determination of physical and chemical properties.

### 2.2. Physicochemical analysis of compost samples

The temperature of the compost was determined as follows: A mercury thermometer was inserted into the heap (80 cm from the ground), the middle (50 cm from the ground), and the lower (20 cm from the ground) parts. The average of the three temperatures was regarded as the final value. The total organic carbon (TOC) and total nitrogen (TN) were analyzed using an Element Vario MACRO cube (Elementar, Germany; Chen et al., 2019). The total phosphorus (TP) and total potassium (TK) were analyzed *via* the molybdenum blue colorimetric method and a flame spectrophotometer (Zhang et al., 2018). The humic acid (HA) and fulvic acid (FA) levels were analyzed as detailed in previous research (Ye et al., 2021; Yang et al., 2023).

### 2.3. DNA extraction and high-throughput sequencing

The total DNA was extracted from a 0.25 g fresh sample by using a FastDNA SPIN Kit for Soil (MP Biomedicals, United States), as per the manufacturer’s instructions. Then, the DNA was quantified using a NanoDrop 2000 (Thermo, United States) and stored at − 20°C before high-throughput sequencing.

The V3–V4 region of the bacterial 16S rRNA gene was amplified with the primers 338F (5′-ACTCCTACGGGAGGCAGCAG-3′) and 806R (5′-GGACTACHVGGGTWTCTAAT-3′). Polymerase Chain Reaction (PCR) amplification was performed in a total volume of 25 μl of reaction mixture with T100 Thermal Cycler (Bio-rad, United States) containing 25 ng of template DNA, 12.5 μl of 2 × T5 Super PCR Mix (Tsingke, China), 1 μl of each primer, and double distilled water to adjust the final volume. All the PCR samples were purified using an E.Z.N.A Gel Extraction Kit D2501 (OMEGA, United States), and the concentration of DNA was determined using the QuantiFluor-ST system (Promega, United States).

The sequencing was performed on the Illumina MiSeq platform. The sequences with similarity of ≥ 97% were assigned to an operational taxonomic unit (OTU). Alpha and beta diversities were applied to analyze the complexity and diversity of bacteria. To identify the difference and similarity of the bacterial community among all samples of the three treatments, principal coordinate analysis (PCoA) was performed, and the significant differences were evaluated by analysis of similarities (ANOSIM). Redundancy analysis (RDA) was performed using the OmicShare tools.^1^ Pearson’s correlations among the bacterial and environmental factors were determined using R.

### 2.4. Molecular ecology network

To understand the interactions among the various bacteria, the phylogenetic molecular ecology network (pMEN) of the bacterial community was analyzed using the Molecular Ecological Network Analysis (MENA) pipeline,^2^ and Cytoscape was used for graphics (Shannon et al., 2003; Zhou et al., 2010; Deng et al., 2012). The module was built up by the greedy optimization method, as described in previous research (Deng et al., 2012). The nodes and edges in the network represented the OTUs and the correlations between OTUs, respectively. The size of a node represents the number of connections between the OTU and other OTUs. The nodes in the same module are more closely connected than those nodes not in the same module.

### 2.5. Database and statistical analysis

The original Miseq sequence data were deposited in the SRA database at the NCBI under accession numbers PRJNA922213 and PRJNA933276.

Each analysis was conducted in triplicate at least. The data were processed using SPSS statistics 25 (IBM, United States), while the significance of the differences was tested using the AVAON test at *p <* 0.05. Figures were generated using Microsoft PowerPoint (Microsoft, United States) and R software 4.0.2.

## 3. Results

### 3.1. Changes in the physicochemical characteristics of the compost samples

**Figure 1:**
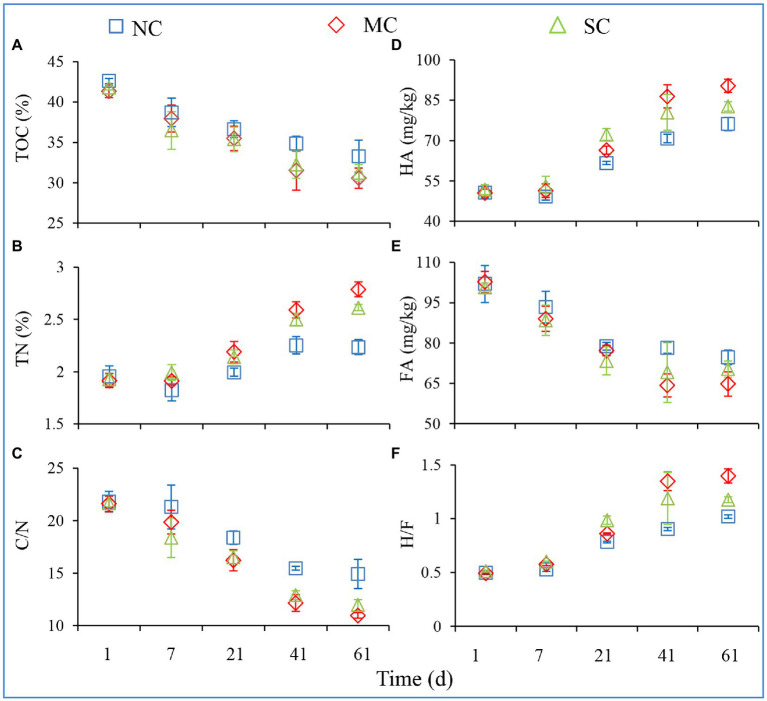
Changes in physicochemical characteristics during the composting process. TOC **(A)**, TN **(B)**, HA **(D)**, and FA **(E)** denote the total organic carbon, total nitrogen, humic acid, and fulvic acid, respectively. C/N **(C)** and H/F **(F)** denote the TOC-to-TN ratio and HA-to-FA ratio, respectively. The error bars represent the standard deviations of the means.

The total composting cycle lasted for about 60 days. The temperature, TOC, TN, TOC/TN (C/N), TP, HA, and FA of all treatments on Days 1, 7, 21, 41, and 61 were detected during composting; these can reflect the composting efficiency and are shown in Figure 1 and Supplementary Figure S1. During the composting process, there was no significant difference in the contents of TOC, TP, and FA in the three different treatments (Figures 1A,E; Supplementary Figure S1B). Meanwhile, the contents of TN and HA in the MC were significantly higher than those in the NC (ANOVA; *p* < 0.05) on Day 61, increasing by 125 and 119%, respectively. The contents of TN in the NC, MC, and SC were 2.24, 2.79, and 2.61%, respectively (Figure 1B). Amendment with matured compost significantly reduced the C/N ratio of the compost, indicating that it may promote the maturity of compost (Figure 1C). The contents of HA increased from 50.64 to 76.14 g/kg, from 50.57 to 90.38 g/kg, and from 51.75 to 82.70 g/kg during composting in the NC, MC, and SC, respectively (Figure 1D). On the contrary, the contents of FA decreased in all treatments from Day 1 to Day 61. Furthermore, on Day 61, the content of HA in MC treatments was significantly higher than that in NC and SC, leading to the highest humic acid/fulvic acid ratio, indicating that matured compost amendment improved the efficiency of organic matter conversion to humic acid and is beneficial to compost maturity (Figure. 1E).

### 3.2. Variability of microbial community diversity across composting

**Figure 2:**
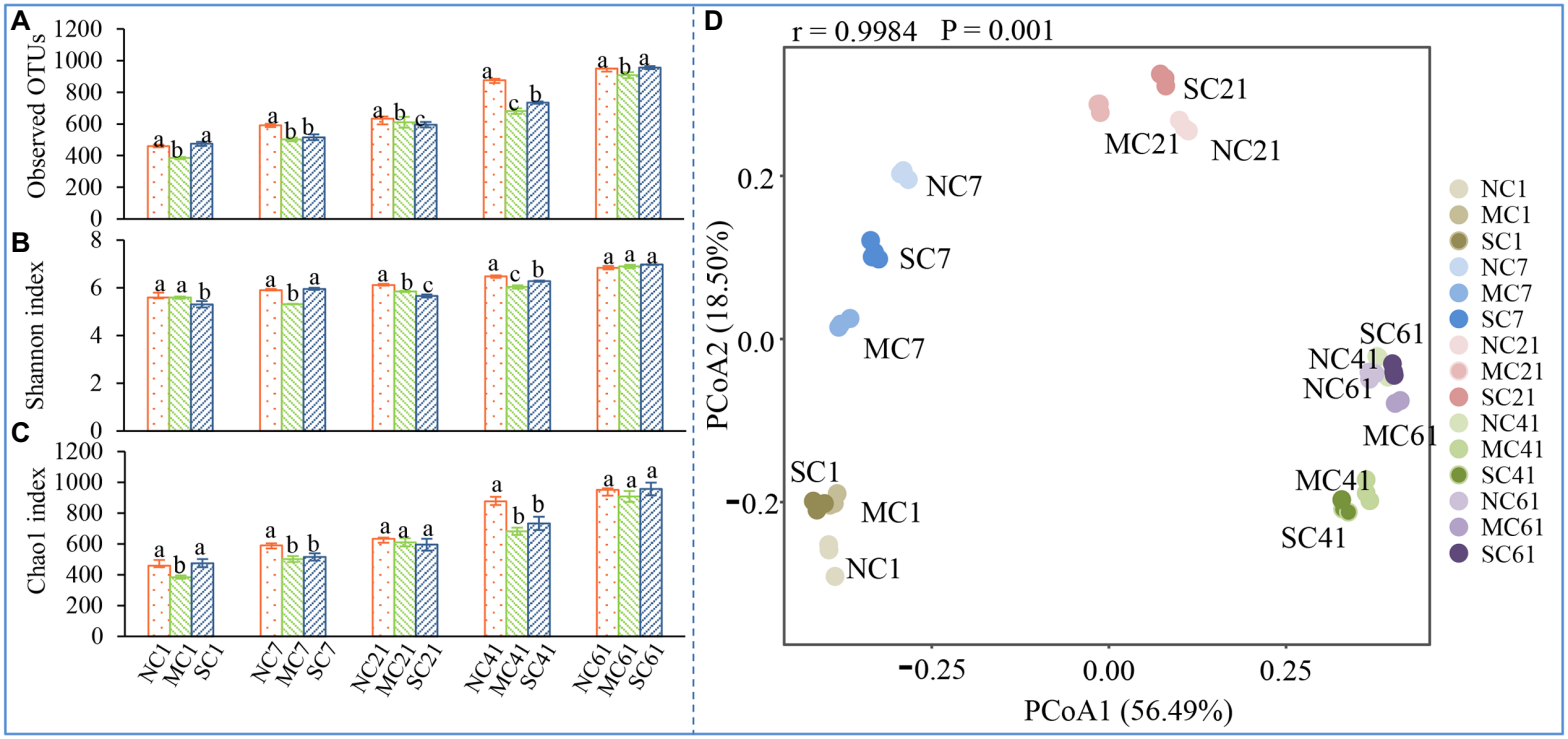
Changes in the Observed OTUs **(A)**, Shannon index **(B)**, Chao1 index **(C)**, and principal coordinate analysis of the bacterial community **(D)**. Different letters denote significant differences by ANOVA test (*p* < 0.05). The error bars represent the standard deviations of the means.

To explore the diversity and structure of bacteria in the three different treatments during composting, 16S rRNA gene high-throughput sequencing was used in this study. After assembly and quality filtering, high-quality sequences were obtained at last. As shown in Figures 2A–C, the alpha indices, including the Observed OTUs, Shannon index, and Chao1 index of the MC, were almost all lower than those of the NC during composting. In particular, on Days 7 and 41, the Observed OTUs, Shannon index, and Chao1 index of the MC were significantly lower than those of the NC (ANOVA, *p* < 0.05), indicating that amendment with matured compost reduced the bacterial community diversity during composting in our research. Moreover, with the composting process, the alpha index values increased gradually from Days 1 to 61 in all treatments. On Day 61, the Observed OTUs index values of the NC, MC, and SC were 950.00, 906.40, and 956.19, respectively—2.07, 2.36, and 2.01 times those on Day 1. A similar result was also observed in the Chao1 index, indicating that the diversity of the bacterial community increased as composting progressed.

The changes in bacterial community structure at the beta diversity level were depicted in the PCoA plot. In Figure 2D, the horizontal and vertical axes show the two principal components of influence, referred to as PCo1 and PCo2, which explain 56.49 and 18.50% of the variance, respectively. The PCoA plot shows that there were huge differences in bacterial communities in different composting periods. On Days 7, 21, and 41, the differences in PCo1 and PCo2 between the NC and MC were more significant than those on Days 1 and 61 (ANOSIM, *p* < 0.05), indicating that amendment with matured compost may have a great impact on the bacterial community structure of compost during the mesophilic phase, thermophilic phase, and cooling phase. However, on Days 1 and 61, no significant difference was observed, indicating that similar bacterial communities were observed during the initial phase and mature phase. These observations show that matured compost amendment had a huge impact on the bacterial community from the mesophilic phase to the cooling phase during composting.

### 3.3. Detailed changes in bacterial abundance across composting

**Figure 3:**
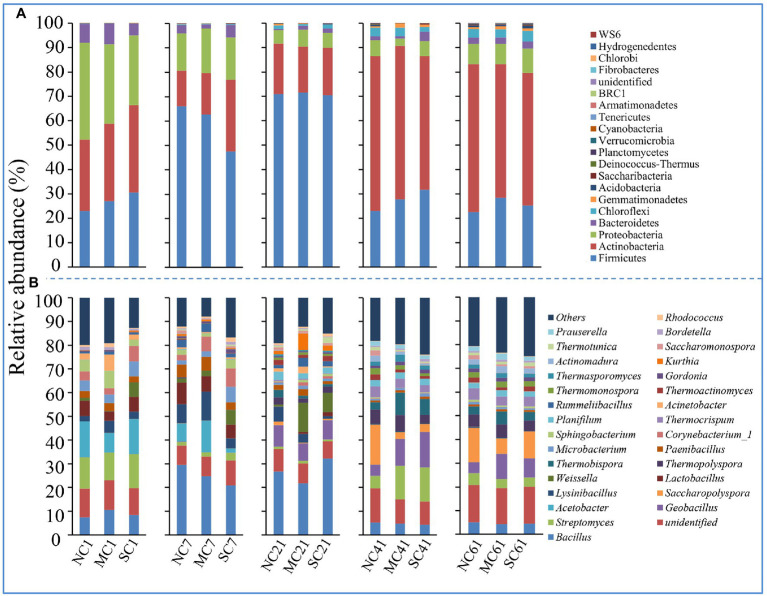
Relative abundance of bacterial species at the phylum **(A)** and genus **(B)** levels.

To further understand which phylotypes were affected by the matured compost amendment, the relative abundances of main bacteria at the phylum level and genus level were summarized (Figure 3). Firmicutes was the most abundant bacteria during composting, ranging from 22.63 to 71.54%, followed by Actinobacteria (14.53–64.44%), Proteobacteria (2.99–39.77%), and Bacteroidetes (1.58–8.54%). At the genus level, *Bacillus* (4.27–32.22%), *unidentified* (7.22–15.81%), *Streptomyces* (0.86–14.44%), *Geobacillus* (0.01–15.05%), *Acetobacter* (0.00–15.14%), *Saccharopolyspora* (0.01–12.17%), and *Lysinibacillus* (0.22–12.17%) were the main genera (for which the maximum abundance was greater than 10%) during composting in our research (Figure 3A). As shown in Supplementary Tables S1 and S2, the main phyla of matured compost which was added to the MC were Firmicutes (54.13%) and Actinobacteria (45.31%), and the main genera were *Corynebacterium*_1 (43.98%), *Kurthia* (28.18%), and Aerococcus (14.76%).

When it comes to different composting phases, amendment with matured compost affects the relative abundance of bacteria at the different taxonomic levels. As shown in Figure 3A, at the phylum level, the relative abundance levels of Firmicutes at the mesophilic phase and thermophilic phase were significantly higher than those at the initial phase, cooling phase, and mature phase, indicating that the Firmicutes may be more adaptable to a higher temperature during composting. Moreover, amendment with matured compost had little effect on the bacterial community at the phylum level.

**Figure 4:**
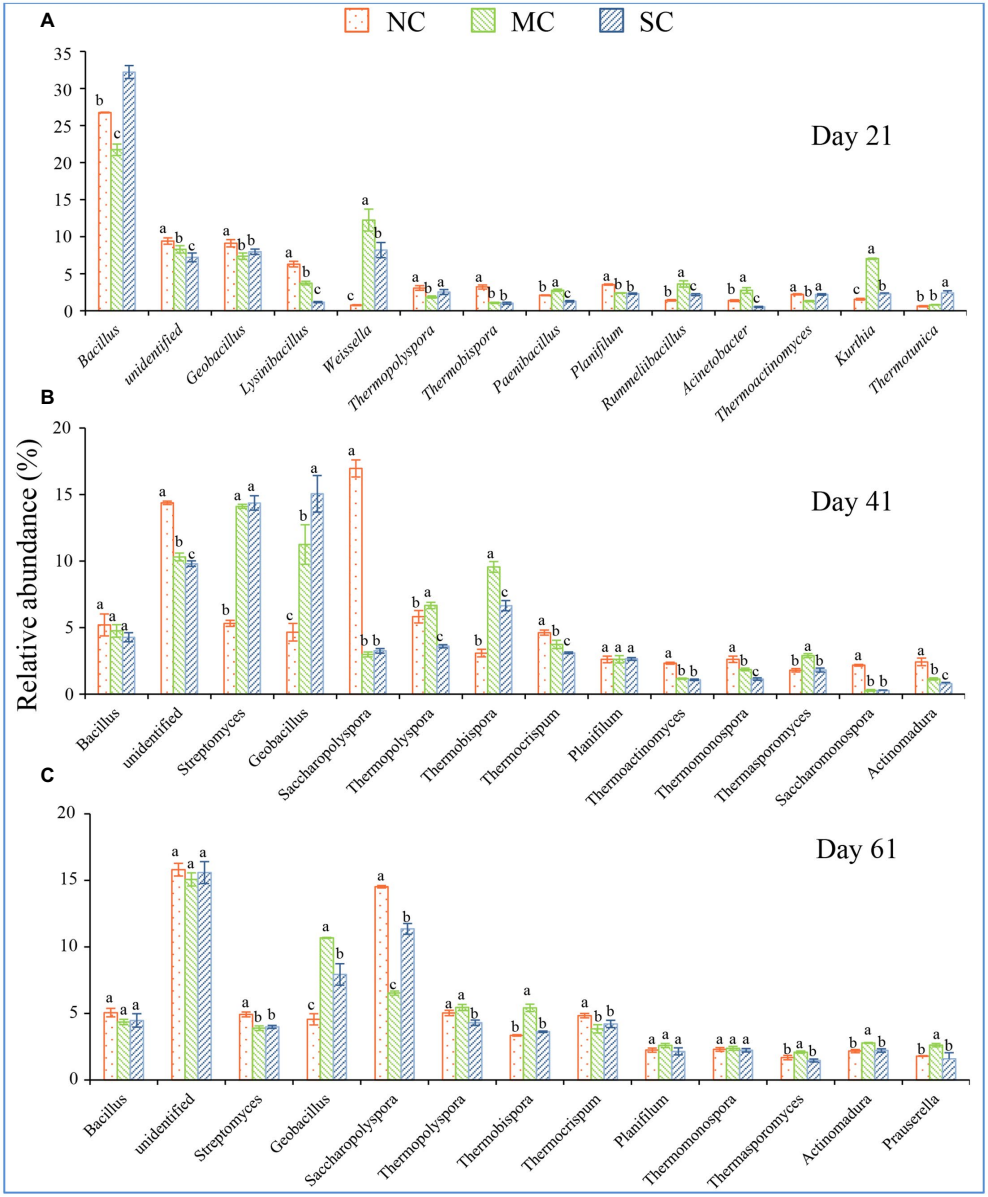
The dominant community changes in the different phases of the composting process. **(A–C)** shows the relative abundance of dominant genera on Days 21, 41, and 61, respectively. The error bars represent the standard deviations of the means. Different letters denote significant differences by ANOVA test (*p* < 0.05).

To further analyze the changes in the bacterial community at the genus level, we selected the dominant community (with maximum relative abundance of > 2%) in the composting process, and we found that amendment with matured compost could change the dominant community in the composting process at the low taxonomic level (Figure 3B). At the initial phase of composting, compared with the other two treatments, amendment with matured compost significantly improved the relative abundance levels of *Acinetobacter* (6.84%), *Sphingobacterium* (7.29%), *Lysinibacillus* (5.01%), and *Bacillus* (10.52%; Supplementary Figure S3A). At the mesophilic phase (7 days), amendment with matured compost significantly improved the relative abundance levels of *Lysinibacillus* (12.17%) and *Acetobacter* (13.26%; Supplementary Figure S3B). At the thermophilic phase (on Day 21), the addition of matured compost significantly improved the relative abundance levels of *Kurthia* (7.04%), *Acinetobacter* (2.76%), *Rummeliibacillus* (3.62%), *Paenibacillus* (2.79%), and *Weissella* (12.23%; Figure 4A). At the cooling phase, the relative abundance levels of *Thermopolyspora*, *Thermobispora*, and *Thermosporomyces* were significantly higher than those for the other two treatments (Figure 4B). Finally, at the mature stage, the relative abundance levels of *Geobacillus* (10.68%), *Thermopolyspora* (5.45%), *Thermobispora* (5.43%), *Actinomadura* (2.79%), and *Prauserella* (2.63%) were significantly higher than those for the other two treatments (Figure 4C). In our research, amendment with matured compost increased the levels of thermophilic bacteria at the cooling phase, including *Thermopolyspora*, *Thermobispora*, and *Thermosporomyces*. There were more thermophilic bacteria at the mature phase in all three treatments than at the initial phase (Supplementary Figure S3B).

### 3.4. The links among bacterial communities and numerous environmental factors

**Figure 5:**
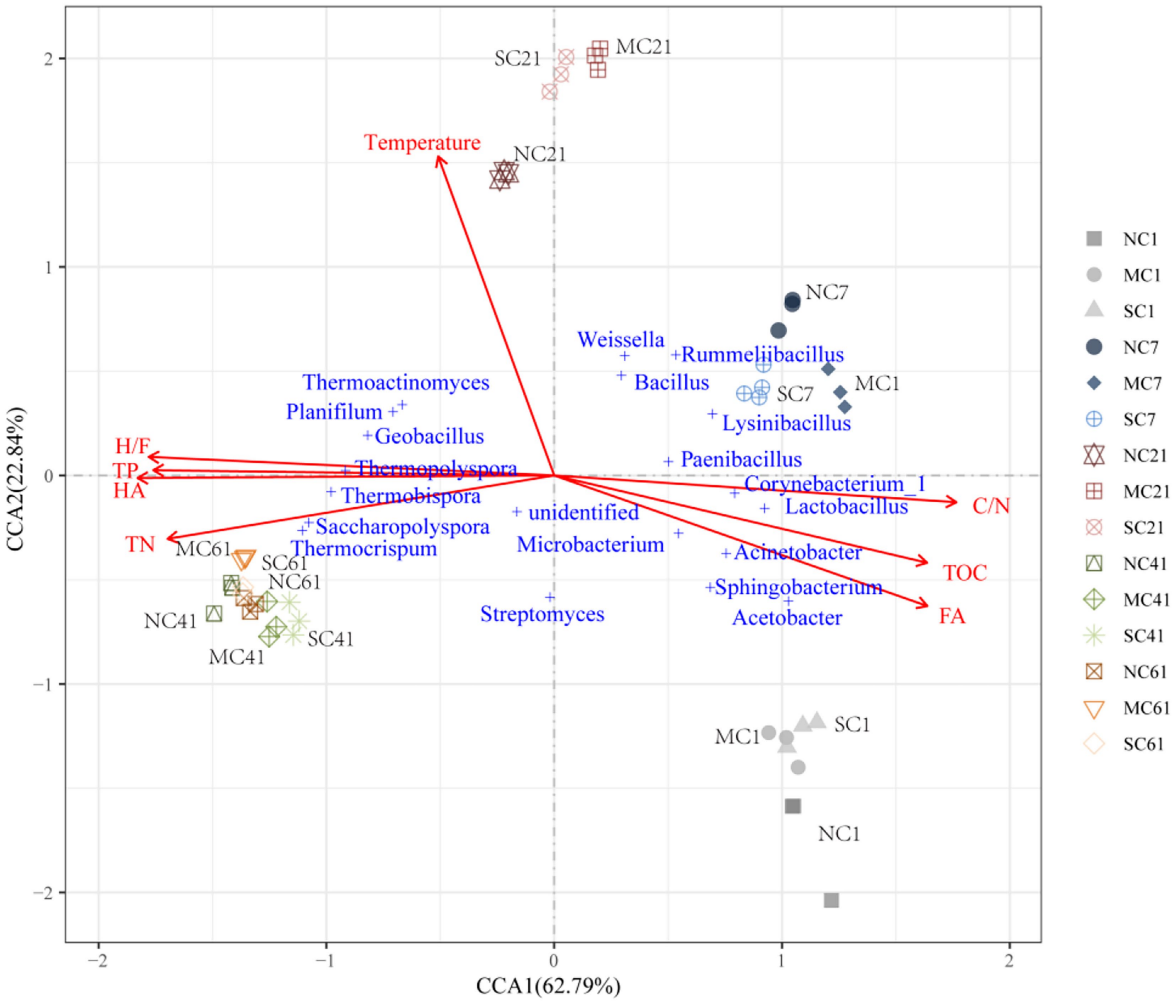
Canonical correspondence analysis of the bacterial community and environmental factors during composting. TOC, TN, HA, and FA denote the total organic carbon, total nitrogen, humic acid, and fulvic acid, respectively. C/N and H/F denote the TOC-to-TN ratio and HA-to-FA ratio, respectively.

Canonical correlation analysis (CCA) was used to analyze the relationships among environmental factors, including TOC, TN, C/N, TP, HA, FA, H/F, and temperature, and the bacterial community at the genus level. The CCA1 axis and CCA2 axis explained 62.79 and 22.84% of the variation during composting, respectively. As shown in Figure 5, the angle between the change trend of the bacterial community structure from Day 1 to 21 and the ray where the temperature is located was the smallest, indicating that the temperature change in Days 1–21 may be the main reason for the change in the bacterial community structure. According to the angle between physical and chemical properties and bacterial genera, we found that bacteria of different genera in the sample had different responses to physical and chemical properties. The TOC and C/N ratio had a negative impact on the thermoduric bacteria, including *Thermoactinomyces*, *Thermopolyspora*, *Thermobispora*, and *Thermocrispum*.

Correlation analysis between thermoduric bacteria and TOC showed that TOC was significantly negatively correlated with *Thermopolyspora* and *Thermocrispum*. Meanwhile, the angle between the ray representing the TOC or C/N ratio and the ray representing TN, TP, HA, and the HA/FA ratio is close to 180 degrees, indicating that they were negatively correlated. Seven genera of bacteria, including *Acetobacter*, *Lysinibacillus*, and *Lactobacillus*, were all positively correlated with the HA content.

### 3.5. Dynamics of the dominant microbes during composting

**Figure 6:**
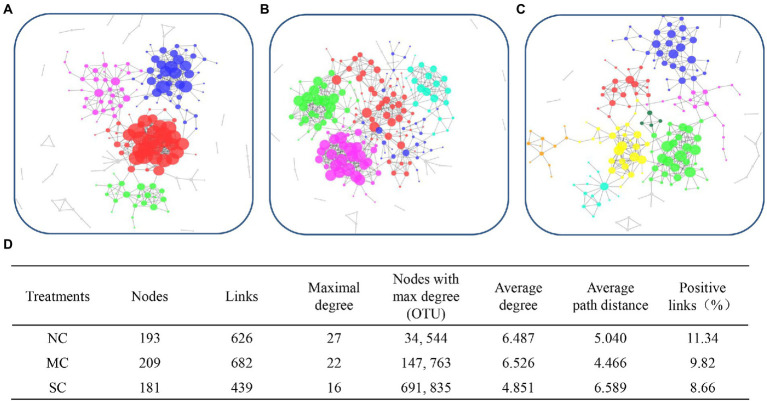
The network interactions of microbial communities in the treatments NC **(A)**, MC **(B)**, and SC **(C)**, and detailed parameters of the networks **(D)**.

To explore the interaction model of the bacterial community during composting, MENA was used to analyze the co-occurrence network. As shown in Figures 6A–D, there were 4, 5, and 8 modules; 193, 209, and 181 nodes; and 626, 682, and 439 links in the NC, MC, and SC, respectively, indicating that amendment with matured compost can change the interaction mode of bacteria during the composting process. The higher numbers of modules, nodes, and links observed in the MC indicates that amendment with matured compost improved the complexity of the co-occurrence network during the composting process.

Furthermore, the nodes with the maximal degrees were also regarded as the keystone species of the bacterial community. As shown in Figure 6D, the keystone species of the NC were OTU34 and OTU 544, belonging to *Acinetobacter* and *Sphingobacterium*, respectively. The keystone species of the MC were OTU 147 (*Comamonas*) and 763 (*Streptomyces*), different from those of the NC, indicating that amendment with matured compost change the core microorganisms. Furthermore, there was no network hub in the NC and MC. OTU 950 (*Thermobispora*) and OTU 1031 (*Streptosporangiaceae*), for which Pi > 2.5 and Zi < 0.62, were the module hubs of the bacterial community in the NC. Meanwhile, OTU 136, which belongs to *Stenotrophomonas*, was the module hub of the MC.

The average path distance and average degree, which both reflect the closeness of the relationship among microbes, were lower for the MC than for the NC, indicating that amendment with matured compost led to a stronger bond among different species of bacteria. However, the smallest percentage of positive links among bacteria was observed in the MC. As shown in Supplementary Table S3, the nodes in Figures 6A–C were analyzed at the phylum level. The main OTUs which the nodes belonged to were Actinobacteria, Bacteroidetes, Firmicutes, and Proteobacteria at the phylum level in our research. The abundance of nodes belonging to *Firmicutes* in the MC (88) was higher than that in the NC (76).

## 4. Discussion

### 4.1. Amendment with matured compost improved the humic acid and nitrogen contents

The main source of humic substances, which can be mainly divided into humic acid and fulvic acid, is the decomposition of lignin, cellulose, and polysaccharide (Pozdnyakov et al., 2020). As shown in Figure 1D, the HA level in the MC was higher than that in the NC. The increased HA in the MC may be produced by cellulose-degrading bacteria. As shown in Supplementary Table S4, on Days 21 and 41, the contents of cellulose in the NC were higher than those in the MC, indicating that the rapid degradation of cellulose may be the direct reason for the increase in the humic acid content in the MC. Previous research clarified that the addition of high-temperature cellulose-degrading bacteria during composting could accelerate the degradation of lignin and cellulose and increase the production of humic acid (Wu et al., 2017; Guo et al., 2019). During composting, humic substances are formed during the cooling and maturation process, and the precursors of humic substances are formed during the thermophilic phase. In our research, on Days 41 and 61, which referred to the cooling and maturation process, the contents of HA in the MC treatment were the highest, reaching 86.43 and 90.38 g/kg; these were significantly higher than those in the NC (ANOVA; *p* < 0.05). A few studies have shown that adding microbial agents can promote the synthesis of humic substances. Similar results were observed by other researchers: Jiang et al. (2015) and Zhang et al. (2018) both found that adding functional microbes accelerates the maturation of compost and promotes the formation of humic substances. The increase in the HA/FA ratio during composting can be attributed to the activity of enzymes that can degrade organic matter, including cellulose, hemicellulose, and ligin (Awasthi et al., 2018). The content of cellulose in the MC was significantly lower than that in the NC. Further, some bacteria, including *Thermopolyspora*, *Thermobispora*, *Thermocrispum*, etc., which have all been identified as being involved in cellulose degradation, were negatively correlated with cellulose contents (Table 1).

**Table 1:** Pearson’s correlation analysis between the concentration of cellulose or hemicellulose and the relative abundance of bacteria at the genus level.

Genus	Cellulose	Hemicellulose
*Bacillus*	0.567^*^	−0.144
*Saccharopolyspora*	−0.606^*^	−0.219
*Lactobacillus*	0.572^*^	0.390
*Weissella*	0.633^*^	−0.040
*Thermopolyspora*	−0.743^**^	−0.457
*Thermobispora*	−0.773^**^	−0.433
*Microbacterium*	0.545^*^	0.634^*^
*Sphingobacterium*	0.561^*^	0.563^*^
*Thermocrispum*	−0.810^**^	−0.328
*Planifilum*	−0.485	−0.473
*Acinetobacter*	0.528^*^	0.316
*Thermomonospora*	−0.688^**^	−0.390
*Gordonia*	−0.757^**^	−0.214
*Thermasporomyces*	−0.797^**^	−0.454
*Actinomadura*	−0.700^**^	−0.185
*Bordetella*	0.040	0.556^*^
*Prauserella*	−0.769^**^	−0.233
*Rhodococcus*	0.637^*^	0.282

*p < 0.05; **p < 0.01.

The addition of mature compost increased the final TN content, indicating that mature compost amendment may be an effective way to reduce nitrogen loss during composting. Luo et al. (2014) found that improving the air permeability of the pile by adding mature compost can reduce nitrous oxide levels. Yang et al. (2019) found that mature compost amendment dramatically reduced emissions of ammonia and nitrous oxide by 58.0 and 73.6%, and it ultimately significantly increased the total nitrogen content in the sample. Overall, amendment with matured compost may reduce the nitrogen loss and increase the HA content in CHR composting.

### 4.2. Amendment with matured compost had a huge impact on the bacterial community structure

Amendment with bacteria has been reported as an effective method to change the microbial community structure during composting and to increase the composting temperature (Li et al., 2019; Meng et al., 2019). Temperature is considered one of the most important factors affecting the composting process, and it can affect the microbial community structure and composting efficiency (Ma et al., 2018). The temperature of the NC, MC, and SC reached 55°C or above within 10, 5, and 5 days, respectively. Previous studies reported that inoculation with matured compost and microbes could improve the temperature of peel compost and extend the duration of the thermophilic phase (Ma et al., 2019). Amendment with matured compost also changed the bacterial community in our research. As shown in Figures 2A–C, amendment with matured compost increased the alpha index of the bacterial community. There was a huge difference between the microbial community at the mature phase and that in the initial phase. Ma et al. (2019) also found that matured compost improved the uniformity of the bacterial community and increased the diversity during the mesophilic and thermophilic phases. In our research, amendment with matured compost improved the abundance of thermophilic bacteria (Figures 4B,C; Supplementary Figure S3B). The matured compost had more thermophilic bacteria, including *Bacillus*, *Thermotunica*, etc., and amendment with matured compost meant that a large number of thermophilic bacteria were inoculated into the pile.

In our research, amendment with matured compost affected the co-occurrence network. Previous research showed that inoculation of bacteria and chemicals may all affect the microbial interaction model. Zhang et al. (2021) observed this following the inoculation of *Bacillus* H2 during rice straw composting. The highly connected networks in the MC, which were revealed by the links, average degree, and average path degree, provided more functional redundancy and system stability for the bacterial community in the MC. More negative links were observed in the MC than in other treatments. This might be due to the limited nutrient or resource cascades during composting that may have increased the competition when the matured compost was added (Zhu et al., 2021). The keystone species and module hub of the MC bacterial community structure also changed with matured compost amendment. This observation is consistent with the research of Qiu et al. (2019). In all treatments, the main nodes of the co-occurrence network belonged to Firmicutes, Actinobacteria, and Proteobacteria. *Thermobispora*, *Streptosporangiaceae*, and *Stenotrophomonas* belong to Actinobacteria, Actinobacteria, and Proteobacteria, respectively.

### 4.3. Change in the microbial community structure may be the reason for change in the compost quality

The succession in bacterial communities during the composting produced with or without matured compost was significantly different. Liu et al. (2020) observed that some thermostable bacteria, including *Thermicanus* and *Tepidimicrobium*, strongly contributed to the formation of humic acid during chicken manure composting. In our research, the content of HA was significantly positively correlated with *Thermopolyspora*, *Thermobispora*, *Geobacillus*, etc. (*p* < 0.05), indicating that the formation of humic acid may be closely related to the metabolism of some important microorganisms. Moreover, in our research, as shown in Figures 4A–C, a few cellulose-degrading bacteria, including *Thermopolyspora*, *Thermobispora*, *Thermosporomyces*, etc. (Yeh et al., 2013; Anbarasan et al., 2017; Braga et al., 2021), were observed in the MC at the cooling phase; the abundance of these bacteria was higher than that in the NC. As shown in Figure. S5, the random forest analysis showed that 17 genera were closely related to the formation of humic acid, containing numerous thermotolerant bacteria. The increased abundance of *Thermopolyspora*, *Thermobispora*, and *Thermosporomyces* also had an important, close, and positive effect on the formation of humic acid. This finding may explain the higher humic acid level in the MC, as the degradation of organic matter, such as lignin, cellulose, and hemicellulose, plays a crucial role (Jurado et al., 2015; Zhang and Sun, 2018). Azizi et al. (2021) also found that an enhancement of protease activity associated with the degradation of lignin and cellulose was caused by microbial amendment.

## 5. Conclusion

In conclusion, amendment with matured compost significantly reduced nitrogen loss and enhanced humic acid accumulation during composting as compared with the non-inoculated control. Interestingly, amendment with matured compost affected the bacterial community: it improved the diversity of the bacterial community, increased the complexity of the co-occurrence network, and led to a transition in the keystone and module hub bacteria during composting. The increased abundance of cellulose-degrading bacteria, including *Thermopolyspora*, *Thermobispora*, and *Thermosporomyces*, was significantly higher in the MC than in the NC, and this may contribute to the formation of humic acid. Overall, this study extends our understanding of the effects of matured compost reflux on compost quality and the bacterial community.

## Data availability statement

The data presented in the study are deposited in the SRA repository at the NCBI, accession number PRJNA922213 and PRJNA933276.

## Author contributions

YM: conceptualization, writing–review and editing, and supervision. XS: wrote and revised original draft preparation. CL and DG collected samples and analyzed the data. JL and XG revised the manuscript. All authors contributed to the article and approved the submitted version.

## Funding

This work was supported by the National Natural Science Foundation of China (42207155, 41601264 and 41807047) and the Jiangsu Agricultural Science and Technology Innovation Fund (CX (22)2043).

## Conflict of interest

The authors declare that the research was conducted in the absence of any commercial or financial relationships that could be construed as a potential conflict of interest.

## Publisher’s note

All claims expressed in this article are solely those of the authors and do not necessarily represent those of their affiliated organizations, or those of the publisher, the editors and the reviewers. Any product that may be evaluated in this article, or claim that may be made by its manufacturer, is not guaranteed or endorsed by the publisher.

## Footnotes

^1^ https://www.omicshare.com/tools/Home/Soft/cca

^2^ http://ieg4.rccc.ou.edu/MENA/main.cgi
