# Supplementary material for: Matured compost amendment improves compost nutrient content by changing the bacterial community during the composting of Chinese herb residues
Source: Front Microbiol. 2023 Mar 16;14:1146546. doi: 10.3389/fmicb.2023.1146546 (PMC10060987; doi:10.3389/fmicb.2023.1146546)
Supplement: Supplementary file 1 [file Data_Sheet_1.docx]

**Matured compost amendment improves compost nutrient content by changing the bacterial community during the composting of Chinese herb residues**

Xiuchao Song^1#^, Chao Lu^1#^, Jia Luo ^1,2^, Xin Gong^3^，Dejie Guo^1^, Yan Ma^1,2,*^

**Author affiliation:**

^1^ Institute of Agricultural Resources and Environment, Key Laboratory of Agro-Environment in Downstream of Yangtze Plain, Ministry of Agriculture and Rural, Jiangsu Academy of Agricultural Sciences, Nanjing 210014, China;

^2^National observation & experimental on of Agro-Environment in Luhe, Nanjing, 210014, China;

^3^Jiangsu Collaborative Innovation Center for Solid Organic Waste Resource Utilization & Jiangsu Key Laboratory for Solid Organic Waste Utilization, College of Resources and Environmental Sciences, Nanjing Agricultural University, Nanjing 210095, China

# Xiuchao Song and Chao Lu contributed equally to this work.

**Corresponding author (**Yan Ma**)**

*Phone: +86 25 8439 1805; fax: +86 25 8439 1805; e-mail: myjaas@sina.com. https://orcid.org/

0000-0002-1693-1104


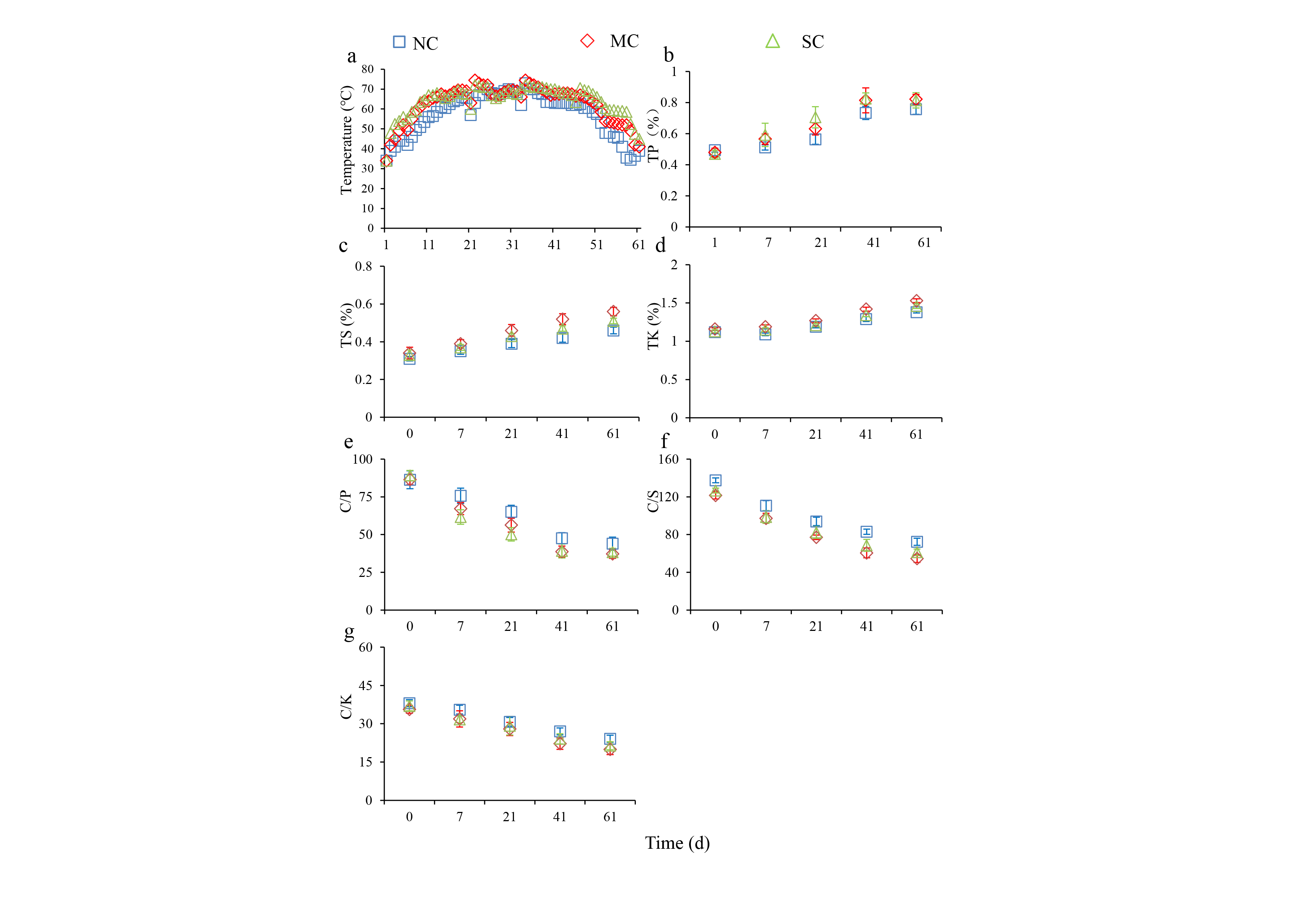


**Fig. S1** The changes of temperature (a),TP (b), TS (c), TK (d), C/P (e), C/S (f), amd C/K (g) during composting. TP, TS, TK, C/P, C/S, and C/K mean the total phosphorus, total sulfur, total phosphorus, TOC-to-TP ratio, TOC-to-TP ratio, amd TOC-to-TK ratio, respectively. The error bars represent the standard deviation of the means.


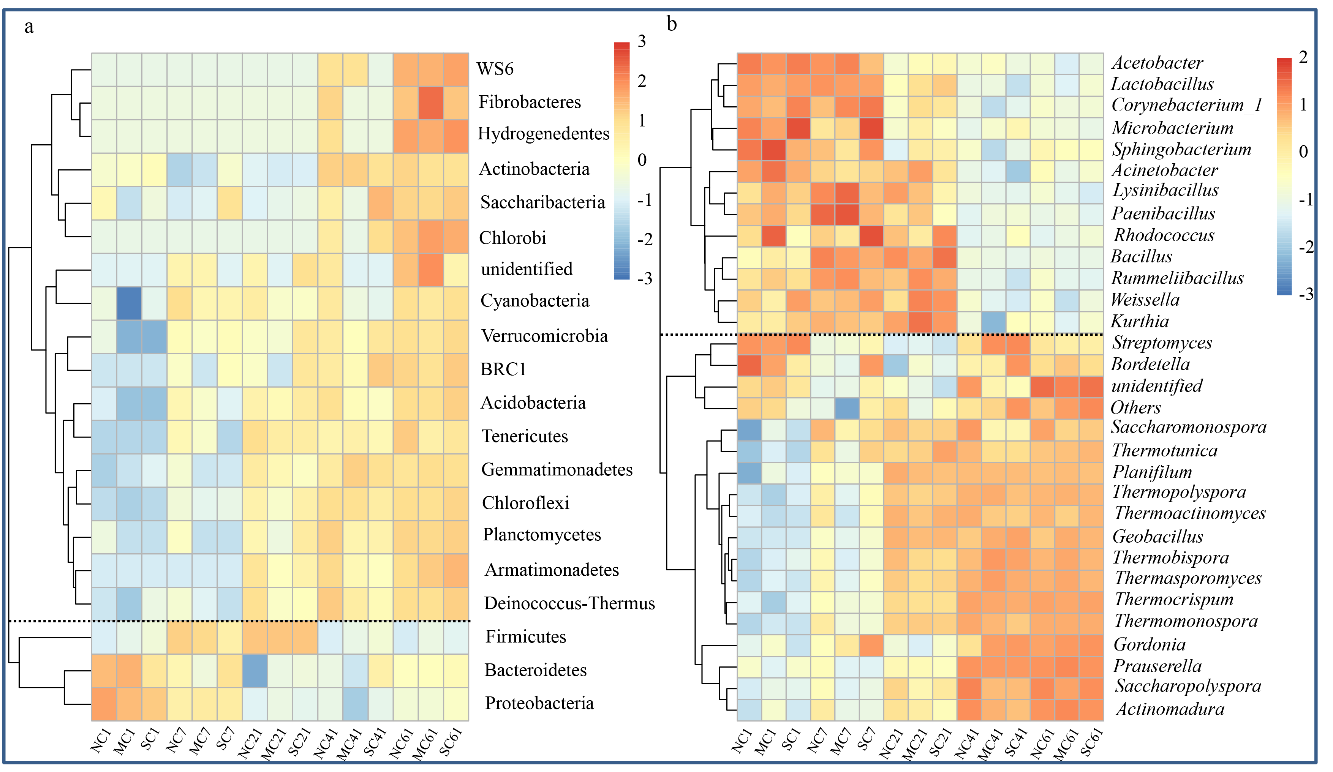


**Fig. S2** Heat map analysis of the bacterial community at the phylum (a) and genus (b) level.


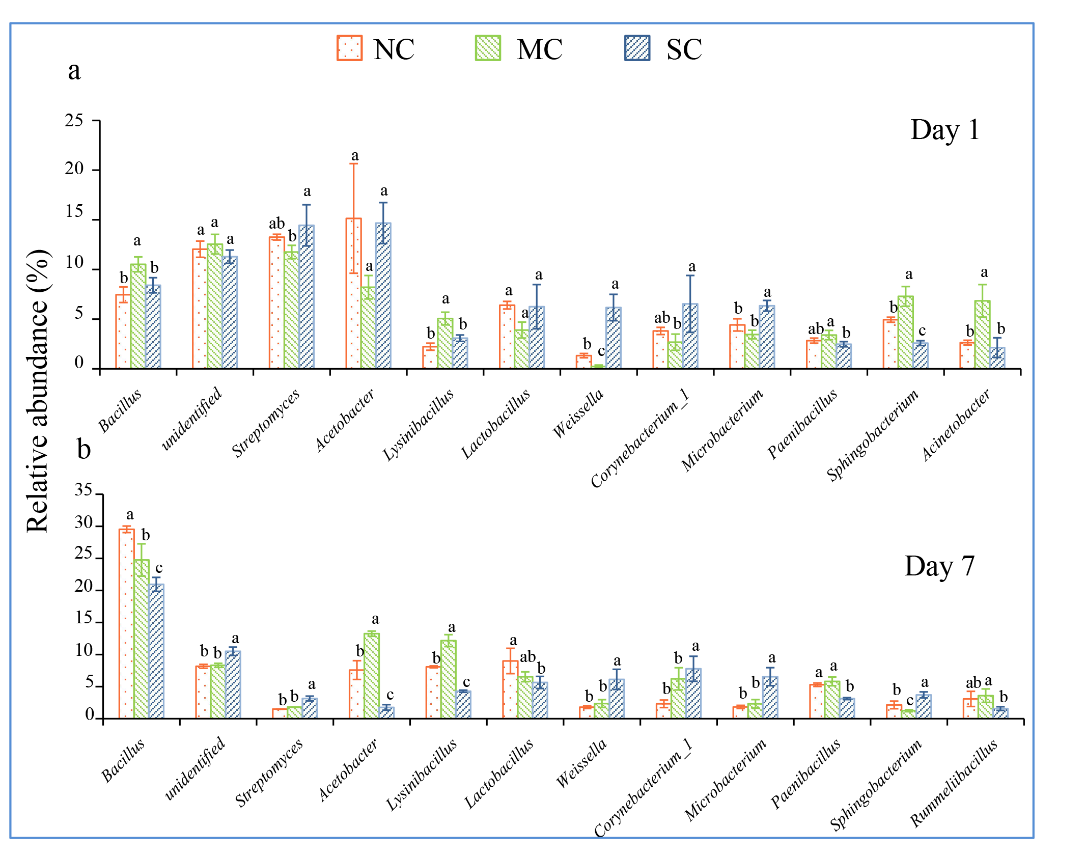


**Fig. S3** The dominant community changes on days 1 and 7. The error bars represent the standard deviation of the means. Different letters denote significant differences by ANOVA test (*P*<0.05).


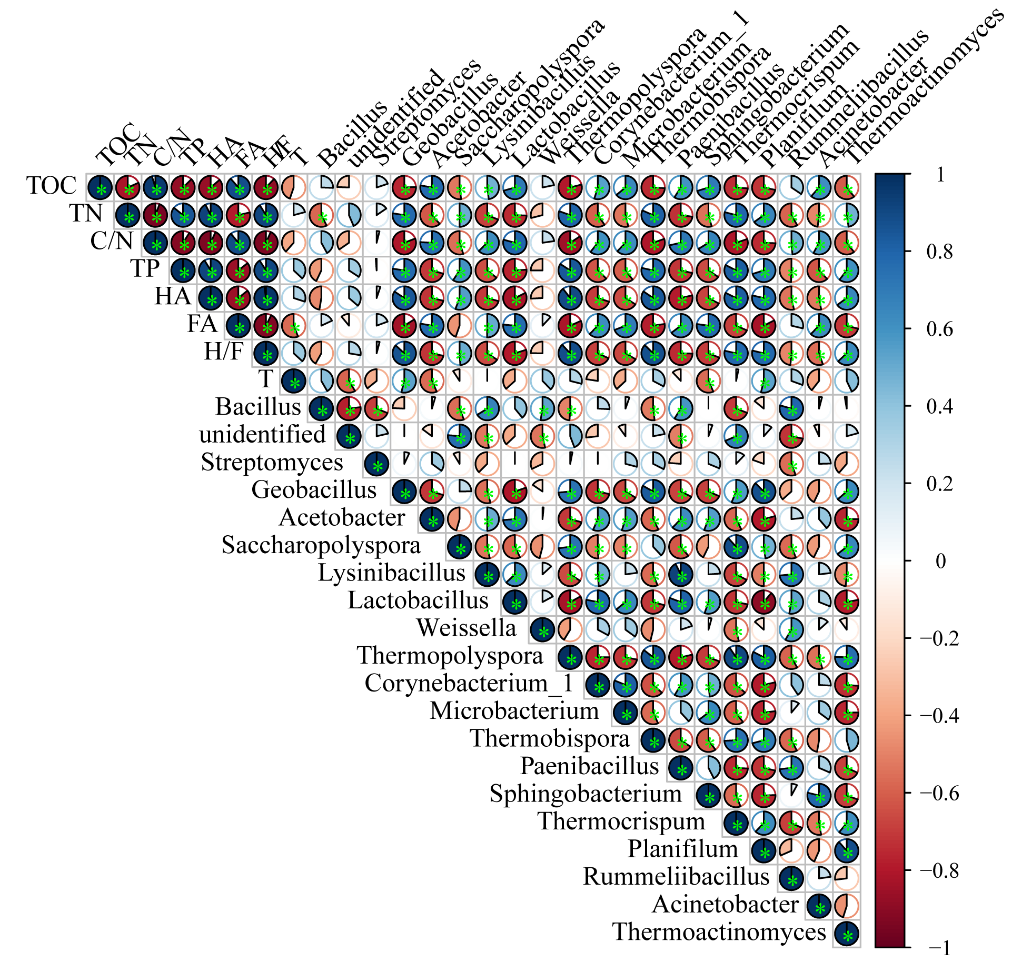


**Fig. S4** Pearson‘s correlation analysis among the environmental factors and the relative abundance of bacteria at the genus level. The green asterisk means that there was extremely significant (*P* < 0.001) correlation between the two factors.


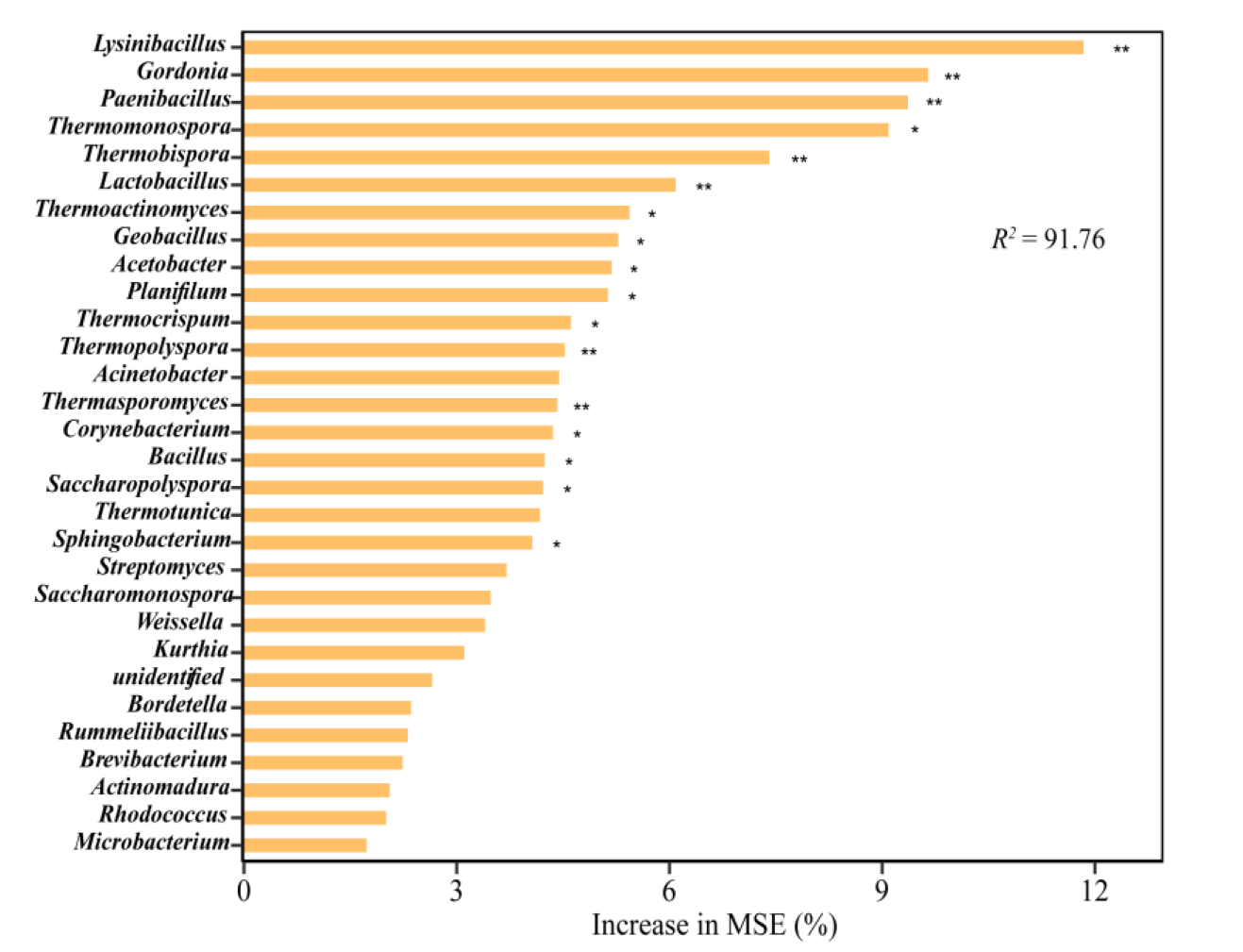


**Fig. S5** The effects of bacterial community at the genus level on the humic acid contents during composting by random forest algorithm. The * and ** represent *P* < 0.05 and 0.01, respectively.

**Table S1** The abundance of bacteria at the phylum level.

| Phylum | Abundance (%) |
| --- | --- |
| Firmicutes | 54.13 |
| Actinobacteria | 45.31 |
| Proteobacteria | 0.33 |
| Bacteroidetes | 0.09 |
| Chloroflexi | 0.08 |
| Thermotogae | 0.03 |
| Tenericutes | 0.02 |

**Table S2** The abundance of bacteria at the genus level.

| Genus | Abundance (%) | Genus | Abundance (%) |
| --- | --- | --- | --- |
| *Corynebacterium_1* | 43.98 | *Atopococcus* | 0.2 |
| *Kurthia* | 28.18 | *Thermotunica* | 0.18 |
| *Aerococcus* | 14.76 | *Brevibacterium* | 0.14 |
| *Bacillus* | 4.1 | *Microbacterium* | 0.14 |
| *unidentified* | 2.87 | *Planifilum* | 0.11 |
| *Desemzia* | 1.58 | *Psychrobacillus* | 0.09 |
| *Lysinibacillus* | 0.7 | *Sphingobacterium* | 0.09 |
| *Rummeliibacillus* | 0.69 | *Pseudomonas* | 0.08 |
| *Oceanobacillus* | 0.55 | *Devosia* | 0.07 |
| *Ureibacillus* | 0.28 | others | 0.96 |
| *Glutamicibacter* | 0.25 |  |  |

| Phylum | Treatment | | |
| --- | --- | --- | --- |
|  | NC | MC | SC |
| Acidobacteria | 1 | 0 | 0 |
| Actinobacteria | 50 | 50 | 52 |
| Bacteroidetes | 15 | 19 | 18 |
| Chloroflexi | 8 | 5 | 5 |
| Cyanobacteria | 1 | 0 | 0 |
| Deinococcus-Thermus | 0 | 1 | 1 |
| Firmicutes | 76 | 88 | 62 |
| Gemmatimonadetes | 2 | 4 | 3 |
| Planctomycetes | 1 | 0 | 0 |
| Proteobacteria | 38 | 40 | 39 |
| Saccharibacteria | 0 | 1 | 0 |
| Verrucomicrobia | 1 | 1 | 1 |

**Table S3** The node numbers of each network at the phylum level.

| Treatment | Cellulose | Hemicellulose |
| --- | --- | --- |
| NC1 | 159.72±1.62 | 81.50±1.15 |
| MC1 | 150.40±1.57 | 56.55±0.42 |
| SC1 | 155.95±0.56 | 69.95±0.63 |
| NC7 | 153.99±0.90 | 46.65±0.50 |
| MC7 | 114.94±0.11 | 37.53±0.27 |
| SC7 | 149.95±1.19 | 62.93±1.10 |
| NC21 | 132.85±1.40 | 54.80±1.03 |
| MC21 | 152.14±1.00 | 35.11±0.78 |
| SC21 | 170.03±1.86 | 49.01±0.39 |
| NC41 | 120.24±0.22 | 35.95±0.18 |
| MC41 | 92.18±1.40 | 32.39±0.27 |
| SC41 | 114.97±2.12 | 41.34±0.11 |
| NC61 | 105.68±1.49 | 49.43±0.16 |
| MC61 | 116.38±0.25 | 58.21±0.21 |
| SC61 | 104.24±1.43 | 58.83±0.86 |

Table. S4 The contents of cellulose and hemicellulose during composting
